# Ensemble Positive Unlabeled Learning for Disease Gene Identification

**DOI:** 10.1371/journal.pone.0097079

**Published:** 2014-05-09

**Authors:** Peng Yang, Xiaoli Li, Hon-Nian Chua, Chee-Keong Kwoh, See-Kiong Ng

**Affiliations:** 1 Data Analytics Department, Institute for Infocomm Research (I2R), Agency for Science, Technology and Research (A*STAR), Singapore, Singapore; 2 Bioinformatics Research Centre, School of Computer Engineering, Nanyang Technological University, Singapore, Singapore; National Institute of Genomic Medicine, Mexico

## Abstract

An increasing number of genes have been experimentally confirmed in recent years as causative genes to various human diseases. The newly available knowledge can be exploited by machine learning methods to discover additional unknown genes that are likely to be associated with diseases. In particular, positive unlabeled learning (PU learning) methods, which require only a positive training set *P* (confirmed disease genes) and an unlabeled set *U* (the unknown candidate genes) instead of a negative training set *N*, have been shown to be effective in uncovering new disease genes in the current scenario. Using only a single source of data for prediction can be susceptible to bias due to incompleteness and noise in the genomic data and a single machine learning predictor prone to bias caused by inherent limitations of individual methods. In this paper, we propose an effective PU learning framework that integrates multiple biological data sources and an ensemble of powerful machine learning classifiers for disease gene identification. Our proposed method integrates data from multiple biological sources for training PU learning classifiers. A novel ensemble-based PU learning method EPU is then used to integrate multiple PU learning classifiers to achieve accurate and robust disease gene predictions. Our evaluation experiments across six disease groups showed that EPU achieved significantly better results compared with various state-of-the-art prediction methods as well as ensemble learning classifiers. Through integrating multiple biological data sources for training and the outputs of an ensemble of PU learning classifiers for prediction, we are able to minimize the potential bias and errors in individual data sources and machine learning algorithms to achieve more accurate and robust disease gene predictions. In the future, our EPU method provides an effective framework to integrate the additional biological and computational resources for better disease gene predictions.

## Introduction

While high-throughput genomic studies have led to the discovery of hundreds and thousands of candidate disease genes, the identification of genes involved in specific human diseases has remained a fundamental challenge, requiring time-consuming and expensive experimentation. Computational approaches that can reliably predict novel disease genes from the vast number of unknown genes will provide a useful alternative to speed up the long and arduous searches for the genetic causes of various human disorders.

Given that an increasing number of genes have been experimentally confirmed over the years as causative genes to various human diseases, it will be useful to develop machine learning methods to identify novel disease genes from the confirmed disease genes as positive training examples, based on the observation that genes associated with similar disease phenotypes are likely to share similar biological characteristics. For example, proteins involved in hereditary diseases tend to be long, with more homologs with distant species, but fewer paralogs within human genome [1]. They are also likely to attach together to form functional modules such as protein complexes [2]. In fact, various studies have shown that genes associated with similar disorders tend to demonstrate similar gene expression profiling [3], high functional similarities [4] [5] and physical interactions between their gene products [6] [7].

In addition, with disease phenotype similarity data, genes associated with same/similar disease phenotypes are likely to share similar biological functions. Given a phonotype *ph_i_*, we can infer its potential disease genes from those disease genes associated with phenotypes *ph_j_* (*ph_i_* and *ph_j_* are very similar) [8].

A number of methods above have thus been proposed to prioritize candidate genes based on different kinds of biological data, such as gene sequence data, gene expression profile, evolutionary features, functional annotation data and PPI dataset. Adie *et al.* [9] employed a decision tree algorithm based on a variety of genomic sequence and evolutionary features, such as coding sequence length and evolutionary conservation, presence, and closeness of paralogs in the human genome. Topological information on PPI network has also been demonstrated to be useful for disease gene prediction. Smalter *et al.* [10] applied support vector machines (SVM) classifier using PPI topological features in addition to sequence-derived and evolutionary features, while Radivojac *et al.* [11] built three individual SVM classifiers using three types of features−PPI network, protein sequence and protein functional information−and then built a final classifier to combine the predictions from three individual classifiers for candidate gene prediction.

The research work mentioned above employed classical machine learning methods to build a binary classifier where the confirmed disease genes are used as positive training set *P* and unknown genes as negative training set *N*. However, these machine learning techniques hardly perform as well as they could because the negative set *N* that they used contained unconfirmed disease genes (false negatives). In light of aforementioned limitation, recently positive unlabeled learning (PU learning) methods have been proposed for the task by building a classification model in which unknown genes are appropriately treated as an unlabeled set *U* (instead of a negative set *N*). For example, Mordelet *et al.* proposed a bagging method ProDiGe for disease gene prediction. It iteratively choosed random subsets (RS) from *U* and then trained multiple classifiers using bias SVM to discriminate *P* from each subset RS. The multiple classifiers were subsequently aggregated to generate the final classifier [12]. Given that the RS's were likely to contain less noise (unknown disease genes) than the original set *U*, it was able to perform better than classical binary classification models that inappropriately used *U* as negative training data. More recently, Yang *et al.* designed a novel multi-level PU learning algorithm PUDI to build a classifier with better performance for disease gene identification where the unlabeled set *U* was partitioned into multiple positive and negative sets with confidence scores for building the classifier [13].

The prior works have clearly shown that integration of various biological data sources is not only desirable but also essential for robust disease gene prediction, since using only a single source of data for prediction is susceptible to incompleteness and noise in the genomic data. It is also advantageous to employ an ensemble approach for prediction, since using a single machine learning predictor is similarly in risk of potential bias caused by inherent limitations of individual prediction models. In this paper, we propose an effective PU learning framework to integrate multiple biological data sources and an ensemble of powerful machine learning classifiers for disease gene identification. In our proposed framework, we first extract multiple positive and negative samples from unlabeled set *U* through performing random walk with restart on different biological networks. We use three biological networks for this paper: protein interaction network, gene expression similarity network, and GO similarity network. Then, we build multiple independent PU learning models that utilize the extracted positive and negative samples as training data with different confidence scores. Finally, we design a novel ensemble strategy called EPU (Ensemble Positive Unlabeled learning) giving optimized weights to base PU learning models to minimize the overall error rate for accurate disease gene predictions.

We compare EPU with multiple state-of-the-art techniques, namely, multi-level example based learning [14], Smalter's method [10], Xu's method [15] and ProDiGe method [12]. The experimental results show that EPU outperforms the existing methods significantly for identifying disease genes on 6 disease groups. In addition, our proposed EPU algorithm also achieves better results when it is compared to three base PU learning classifiers, demonstrating that our proposed ensemble-based approach is able to effectively utilize individual classifiers for better performance. Finally, we also conduct a case study to show how our proposed EPU algorithm can discover novel disease genes for endocrine and cancer diseases.

## Materials and Methods

In this section, we begin with the description of the experimental data used and briefly introduce how the protein interaction network, gene expression similarity network, and GO similarity network [16] [17] [18] are constructed. Then we will present the schema of our proposed EPU algorithm.

### Experimental data and gene network modeling

In this paper, we have exploited the following biological data human protein interaction data, gene expression data, gene ontology, and phenotype-gene association data.

*Human protein interaction data* (PPI) is downloaded from the Human Protein Reference Database (HPRD) [19] and Online Predicted Human Interaction Database (OPHID) [20]. The combined PPI dataset contains 143,939 PPIs among a total of 13,035 human proteins. We build a protein interaction network *G_PPI_* = (*V_PPI_, E_PPI_*) where *V_PPI_* represents the set of vertices (proteins) and *E_PPI_* denotes all edges (detected pairwise interactions between proteins). *G_PPI_* can be represented as its matrix format, i.e. *W_PPI_* = [w*_ij_*] where w*_ij_* = 1 if the corresponding protein pair 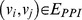; 0 otherwise.

*Gene expression data* is obtained from RNASeq data which is made publicly available in the EBI ArrayExpress, by the Illumina Human BodyMap 2.0 (http://www.ncbi.nlm.nih.gov/geo/query/acc.cgi?acc=GSE30611). The dataset comprises Fastq reads from the paired-end sequencing of cells from 16 human tissue types, including colon, heart, kidney, white blood cells and so on, using the Illumina HiSeq next generation sequencing platform. This dataset represents the expression values of 17,652 human genes on 16 human tissue types. Suppose gene *g_i_* and *g_j_* are represented as their gene expression profile vectors (*x_i_*_1_, *x_i_*_2_,…, *x_in_*) and (*x_j_*_1_, *x_j_*_2_,…, *x_jn_*) respectively where *x_ik_* (*k* = 1, 2, …, *n*) denotes the expression value of gene *i* in the *k*-th tissue. Pearson correlation coefficient is employed to measure the similarity between *g_i_* and *g_j_*:
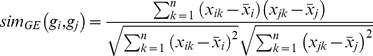
(1)where 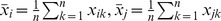.

We build a gene expression similarity network *G_GE_* = (*V_GE_*, *E_GE_*), where 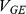 represents a set of genes occurring in the gene expression data and *E_GE_* represents a set of edges between the genes in *V_GE_*. For each gene *g_i_*, we ranked all genes *g_j_* in *V_GE_* where *i≠j* according to decreasing order of *sim_GE_*(*g_i_*, *g_j_*), and add an edge (*g_i_*, *g_j_*) to *E_GE_* if *g_j_* is in the top 5 of the list. This helps to filter low similarity pairs and potential noise in gene expression data. Then we transform the gene expression network *G_GE_* to its matrix format where the edges of two genes are reformatted to their gene expression similarity in equation (1).

*Gene Ontology* (GO, http://www.geneontology.org/) is a set of controlled vocabulary used to annotate genes and gene products [21]. Gene Ontology provides three sub-ontologies, namely, biological process (*BP*), molecular function (*MF*) and cellular components (*CC*) [21]. For each gene, we build a feature vector using its annotations from three sub-onotolgies, i.e. {*MF_1_*,…,*MF_|SMF|_*, *BP_1_*,…,*BP_|SBP|_*, *CC_1_*,…,*CC_|SCC|_*}. For example, a gene *g_i_* is represented as gene vector ***g_i_*** = (*mf_i1_*, …, *mf_i|SMF|_*, *bp_i1_*, …, *bp_i|SBP|_*, *cc_i1_*, …, *cc_i|SCC|_*), where *mf_ij_* (similar for *bp_ij_*, *cc_ij_*) is GO term similarity between *g_i_* and the feature *MF_j_*. Since the GO terms of *BP*, *MF* and *CC* are organized into DAG structure, we use the computational method in [22] to measure the similarity of two GO terms. And |*SMF*| is number of selected MF term features. We choose the GO features that could help distinguish disease genes from non-disease genes with strategy in [13] and top 1000 scored features were selected for each of three feature groups, i.e. *BP*, *MF* and *CC*, respectively. We then build GO similarity network *G_GO_* = (*V_GO_*, *E_GO_*), where *V_GO_* is the gene set annotated in GO dataset and *E_GO_* is a set of edges between the genes in *V_GO_*. Similarly to the gene expression similarity network, we keep those top 5 edges which have highest similarities to each gene and other edges. *G_GO_* can be represented as its matrix format, i.e. *W_GO_* = [*w_ij_*]. Given a gene *g_i_*, if *g_j_* is one of top 5 lists of *g_i_*, *w_ij_* is normalized as:
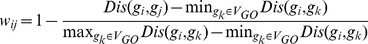
(2)otherwise, *w_ij_* = 0, where *Dis*(*g_i_*, *g_j_*) denotes Euclidean distance between *g_i_* and *g_j_* and 0≤*w_ij_*≤1.

#### Phenotype-gene association data

4260 phenotype-gene association data, spanning 2659 known disease genes and 3200 disease phenotypes, are obtained from the latest version of OMIM (http://omim.org/) [23]. Goh *et al.* [6] have categorized the 3200 disease phenotypes in OMIM database into 22 disease groups/classes, i.e. Cancer, Metabolic, Neurological, Endocrine, etc, based on the physiological system affected. For example, the Endocrine disease group comprises 62 OMIM phenotypes, including OMIM 241850 (Bamforth-Lazarus syndrome) and OMIM 304800 (Diabetes insipidus, nephrogenic) etc.

#### Phenotype similarity network

Disease phenotype similarity network [24], is defined as *G_PH_* = (*V_PH_*, *E_PH_*), where *V_PH_* denotes the set of disease phenotypes and *E_PH_* denotes relevant phenotype pairs. Disease phenotypes in *V_PH_* are represented as feature vectors in which feature terms are Medical Subject Headings (MeSH) controlled vocabulary, and phenotype similarities in *E_PH_* are evaluated underline concept relevance and frequency of MeSH terms appearing in text description of OMIM documents. According to Vanunu *et al.* [8], phenotype pairs with high similarities are regarded as informative and reliable. Therefore, we apply logistic function to filter out low phenotypic similarities in *E_PH_*, following [2] [8].

### The proposed technique EPU

**Figure 1:**
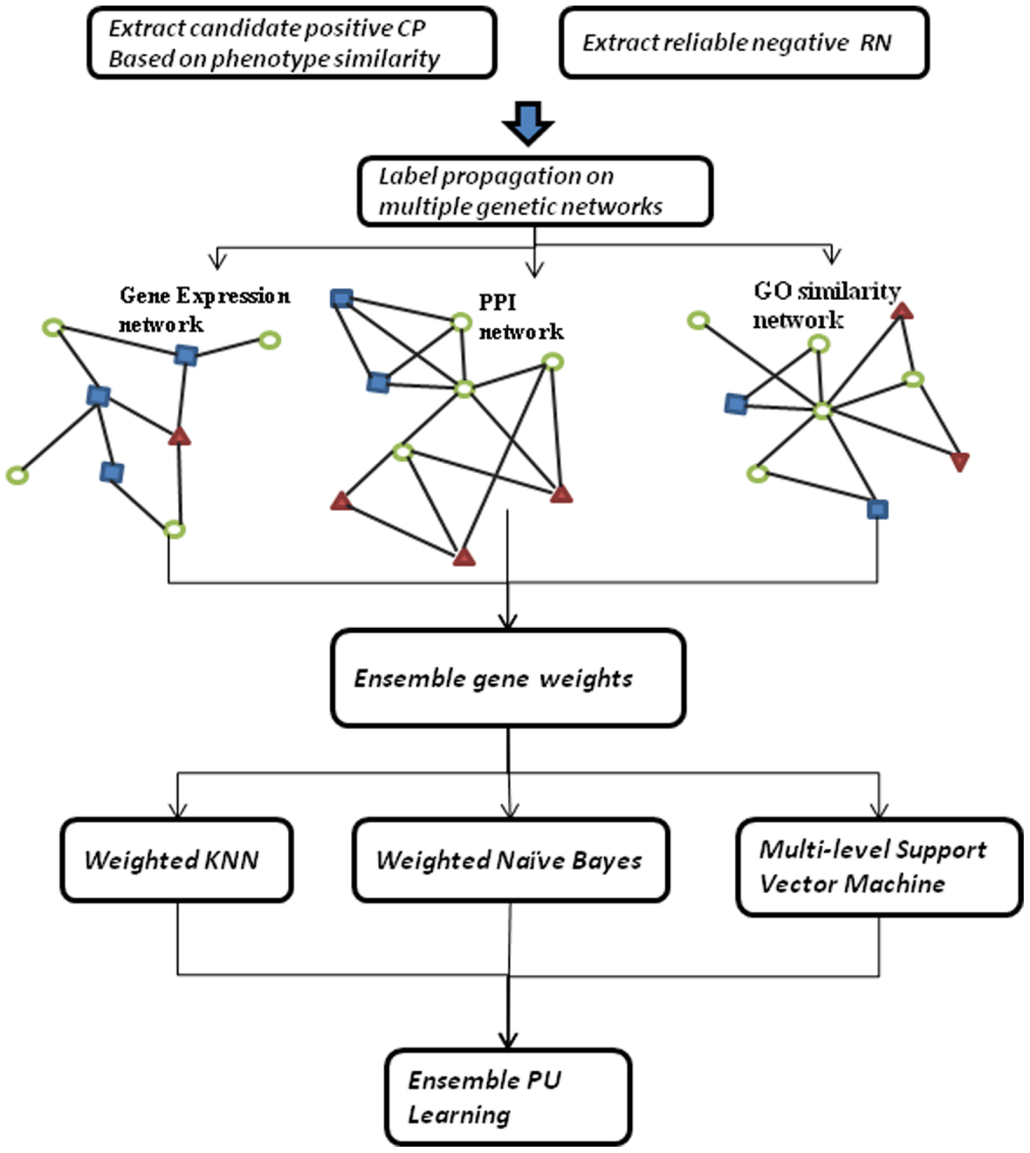
Overall schema of EPU learning algorithm. EPU is a framework that utilizes positive and ‘weighted’ unlabeled examples to build an ensemble classifier for disease gene identification. First of all, EPU extracts candidate positives (CP) and reliable negatives (RN) from unlabeled set. Then it applies random walk algorithm to weight remaining unlabeled genes on genetic networks. To achieve reliable and robust measure on U, EPU consults three biological networks, PPI network, GO similarity network and Gene expression network. After obtained ensemble weighted genes, EPU builds three PU learning classifiers. Finally, a novel ensemble strategy is applied to combines the outputs from these classifiers to make final predictions.

The schema of our EPU algorithm is presented in Figure 1. EPU first selects candidate positives from positive genes and reliable negatives from unlabeled genes. It then builds three gene similarity networks using PPI data, gene expression data and Gene Ontology data and applies random walk on the three networks to propagate weights to unlabeled genes that reflect likelihoods of belonging to positive/negative class. We then exploit the weighted genes to build three diverse classification models to predict “soft” labels for test genes. Finally, an ensemble learning algorithm combines the prediction results from the classifiers to make a final prediction for the classification of the unknown test gene.

Suppose all disease genes from OMIM are stored into a disease gene set *DIS*. All the other genes that are not a member of *DIS* will be treated as unknown/unlabeled genes and be stored into a set *UG* (contains *16,570* genes) [25]. Each gene in *DIS* and *UG* is represented as a feature vector, namely, 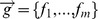 where *m* is the total number of features from GO terms, protein domains and PPI topological features, following our previous work [13].

In the next section, we describe how to predict novel disease genes given the confirmed disease genes for a particular disease or disorder. The confirmed disease genes for the given disorder group are treated as *positive set P* (*P* 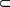 *DIS*) while randomly selected unknown genes from *UG* are treated as *unlabeled set U* (*U* 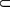 *UG*, |*U*| = |*P*|), following the settings in [9] [10] [15].

### Weighting unlabeled genes by integrating multiple biological evidences

Given a particular disease class and its known associated disease genes, we first build the training data sets for machine learning by prioritizing the candidate positives and reliable negatives based on their similarity to the query disease class. We build three gene similarity networks using PPI, gene expression and GO as described above, and perform a random walk with restart algorithm on these three gene similarity networks to estimate the likelihood of the unlabeled genes belonging to disease class or non-disease class. The details are as follows.

#### Extracting candidate positives and reliable negatives

As a typical positive set *P* is relatively small, we want to find a set of candidate positive genes *CP* to complement *P*. Given that recent studies have shown that similar phenotypes are often caused by functionally related disease genes [4] [6], we could populate the set of candidate positive genes *CP* with genes associated to similar/relevant phenotypes, based on the principle of guilt-by-association. In other words, given a query disease group/class, we can use its associated phenotypes to uncover similar disease phenotypes, as shown in Figure S1.

Having identified the candidate positive genes *CP*, let us now describe how to extract *reliable negative* gene set *RN*. We consider reliable negatives as those unlabeled genes that are very different from positive set *P*. To identify such genes, we first build a “positive representative vector” (*pr*) by summing up gene vectors in *P* and normalizing it. Then, we compute the average *Euclidean distance* [26] of every unlabeled gene *g_i_* in *U* from *pr*. To extract the reliable negative genes for *RN*, we regard an unlabeled gene *g_i_* as a member of *RN* if its distance from *pr* is longer than the average distance (of all the genes in *U*) from *pr*, formalized as follows:
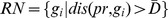
(3)where 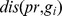 is the Euclidean distance between gene *g_i_* and positive representative vector *pr*. We compute an average distance 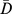 of all the unlabeled gene in *U* from *pr* as: 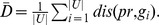


#### Ensemble weighted unlabeled genes via performing label propagation on multiple networks

We now have the given positive set *P*, a candidate positive set *CP*, a reliable negative set *RN* and a remaining unlabeled set 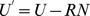 for machine learning. To build a robust classification model, we will extract those genes with reliable labels that are near the decision boundary between the positive and negative classes. We adapt the Random Walk with Restart algorithm [27] to perform flow propagation which spreads the label information from *P*, *CP* and *RN* to the unlabeled genes in 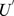 on the biological networks that we have constructed, namely the PPI network *G_PPI_*, the GO similarity network *G_GO_* and the gene expression similarity network *G_GE_* as described earlier.

Formally, let *R_0_* be an initialization vector where primitive scores are assigned to all genes in three networks to indicate the genes' potential classification labels. Let *p_0_*, 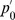 and *n_0_* denote the initial values for genes in *P*, *CP* and *RN* respectively, as follows. The genes 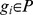 are all given a score *p_0_*(*g_i_*) = +1, indicating their disease gene status. Each candidate positive gene 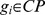 is assigned a score that computes its maximal phenotypic similarity to the known disease genes in *P*,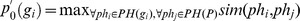, where 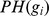 denotes disease phenotypes caused by gene *g_i_*, and *PH*(*P*) denotes disease phenotypes caused by disease set *P*. For genes in reliable negative set *RN*, to balance total amount of flows between positive genes and negative genes, the initial score for negative gene *g_i_* is assigned with
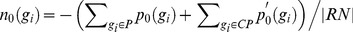
(4)where 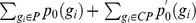 is a total amount of positive gene set. The remaining unlabeled genes in 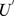 are assigned an initial score of 0.

For each of the three biological networks *G_PPI_*, *G_GE_* and *G_GO_*, prior influence from the seed nodes in *P*, *CP* and *RN* are propagated to their direct neighbors, and then continue to spread to other adjacent nodes iteratively across the network. Given *R_0_* the initial score vector (step 0), *R_t_*, the score vector at step *t*, can be calculated as follows:
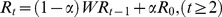
(5)where *R_1_* = *R_0_* and *W* = *D^−1^W* is a normalized format of matrix *W*, 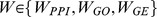. Here *D* is the diagonal matrix and 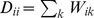. *α* represents the percentage of flow back to original seed nodes in *P*, *CP* and *RN* during each iteration. The default value of 0.7 is used for *α*, following [16].

Eventually, the information flow will converge to a steady state [27]. In our case, the Random Walk with Restart algorithm will stop its iterative process when the difference between two steps *R_t_* and *R_t-1_* is less than 10^−6^ [16], measured by *L1 norm*. The scores for unlabeled genes from the three gene networks are combined into one *integrated score*:
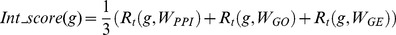
(6)where *R_t_*(*g*,*W_PPI_*), *R_t_*(*g*,*W_GO_*) and *R_t_*(*g*,*W_GE_*) are the scores for gene *g* in the PPI, GO similarity and gene expression similarity networks respectively.

### Ensemble positive unlabeled learning EPU

Next, we describe how to build three separate PU learning classification models Support Vector Machine *SVM*, K-Nearest Neighbor, and Naïve Bayes classifier−to classify genes into two classes *C* = {*+*, *−*}, where ‘*+*’ denotes positive/disease class and ‘*−*’ presents negative/non-disease class.

#### PU learning model 1: Weighted K-Nearest Neighbor (WKNN)

KNN is an instance based learning method, which classifies an unknown test gene based on the class labels of its top *K* nearest training example genes, i.e. based on the majority class vote of its nearest *K* neighbors. The distance between the test gene and other training examples can be computed using common distance metrics such as Euclidean distance. Given a test gene *g_i_* and its *k* nearest neighbor set *D_i_*, we divide *D_i_* into positive and negative training subsets, namely *D_i+_* = {*g|Int_score(g)≥0*, *g*∈*D_i_*} and *D_i-_* = {*g|Int_score(g)<0*, *g*∈*D_i_*} based on these neighbors' integrated scores. The conditional probability of the test gene *g_i_* with respect to disease (+)/non-disease class (−), is measured as
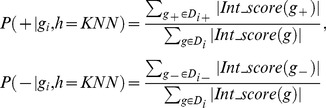
(7)


Note that weighted KNN accumulates both positive and negative integrated scores in *D_i_* and estimates the probability of *g_i_* belonging to positive (or negative) class based on the accumulated scores in that class.

#### PU learning model 2: Weighted Naïve Bayes (WNB)

Given a test gene *g_i_*, the probability that gene *g_i_* belongs to a class *c_j_* (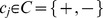) can be computed using Bayes' theorem as:
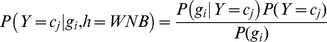
(8)where the probability 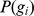 is a constant for the positive and negative classes. Here, we define the prior probabilities of the positive and negative classes as 0.5, i.e. *P*(*Y* = +) = *P*(*Y* = −) = 0.5. Given a gene vector 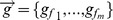, the conditional probability of feature *f_k_* associated with class *c_j_*, denoted as *P*(*f_k_*|*Y* = *c_j_*), is calculated as:
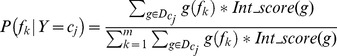
(9)where *g*(*f_k_*) is the value of feature *f_k_* in gene vector 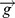, 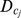
is defined as either 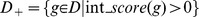 or 
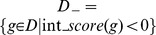
, depending on *c_j_* is positive class (+) or negative class (−).

By assuming that the probabilities of features are independent given the class *Y* = *c_j_*, we obtain the Naïve Bayes classifier:
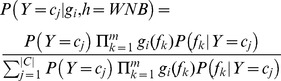
(10)


#### PU learning model 3: Multi-level Support Vector Machine (MSVM)

Based on the integrated score *Int_score*(*g*), we further partition the unlabeled genes 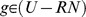 into three parts: likely positive set *LP* (genes get higher positive integrated scores), likely negative set *LN* (genes get lower negative integrated scores) and weak negative set *WN* (remaining genes) using the following criteria:
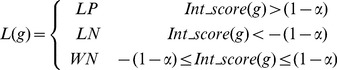
(11)


We then build a multi-level classifier based on positive training set *P*, reliable negative set *RN*, and three newly generated sets *LP*, *LN*, and *WN*, via weighted support vector machine technique [28] [29], to take into account of the inherently different levels of trustworthiness of labels in the five gene set.

The objective function of Weighted SVM can be defined as [14]:
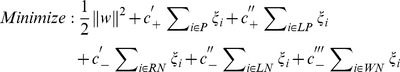
(12)


Subject to: 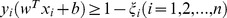 where the values of parameters 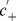, 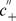, 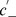, 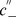 and 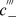 can be decided by using cross-validation techniques. Finally, we apply our MSVM model 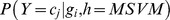 to compute the probability of test gene *g_i_* belonging to class *c_j_* (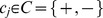) for its classification.

Note that while the candidate positive set *CP* plays a role in assigning the genes in *U* - *RN* to one of the 3 subsets *LP*, *LN* and *WN*, it does not overlap with the training set 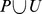 and hence is not used in the construction of the *MSVM* model.

#### Ensemble-based algorithm for integration of individual classifiers

In order to perform more robust classification, we design a novel ensemble learning model to integrate the three classification models constructed above.

Suppose 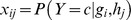 denotes the probability of gene 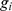 belonging to class *c* as predicted by the *j^th^* classifier. We can organize the genes in *D* in the following matrix:
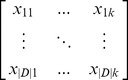
(13)where *k* is the number of individual classifiers (here, *k* = 3), and |*D*| is the size of training set *D*.

Our ensemble model 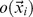 integrates the outputs from the multiple classification models as follows:
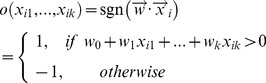
(14)where 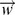 is a weight vector that indicates the importance of individual models. The final output value “1” denotes disease/positive class and ‘−1’ denotes non-disease/negative class.

The classifier weight 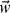 can be learned from training set *D* as follows. We define 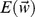 as training error of the hypothesis of our ensemble model:
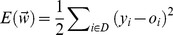
(15)where *y_i_* (*y_i_* 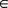
{−1,1}) and *o_i_* (*o_i_* 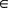
{−1,1}) are the actual class and predicted class by our ensemble model for training gene 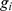 respectively. 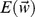 is a linear square error function that evaluates the difference between *y_i_* and *o_i_*. We minimize 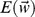 to guarantee the classification output *o* with minimal error rate and calculate the weight vector 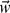.

Here, gradient decent is applied to search the probable weight vectors in error surface. The gradient of *E* for 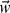, denoted as 
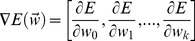
, is the derivative of *E* with respect to each component of the vector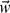. From above equation, we obtain each component of 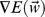 as follows:
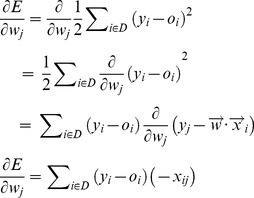
(16)


The following training rule guarantees that 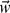 is adjusted in the direction of steepest descent along the error surface: 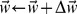, where 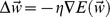. *η* is a small positive constant, called learning rate, to determine the step size in gradient decent exploration. We set *η* = 0.001, following previous work [30]. The negative gradient 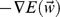 gives the direction of steepest decrease. According to equations above, we update the gradient descent rule as follows:
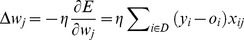
(17)


**Figure 2:**
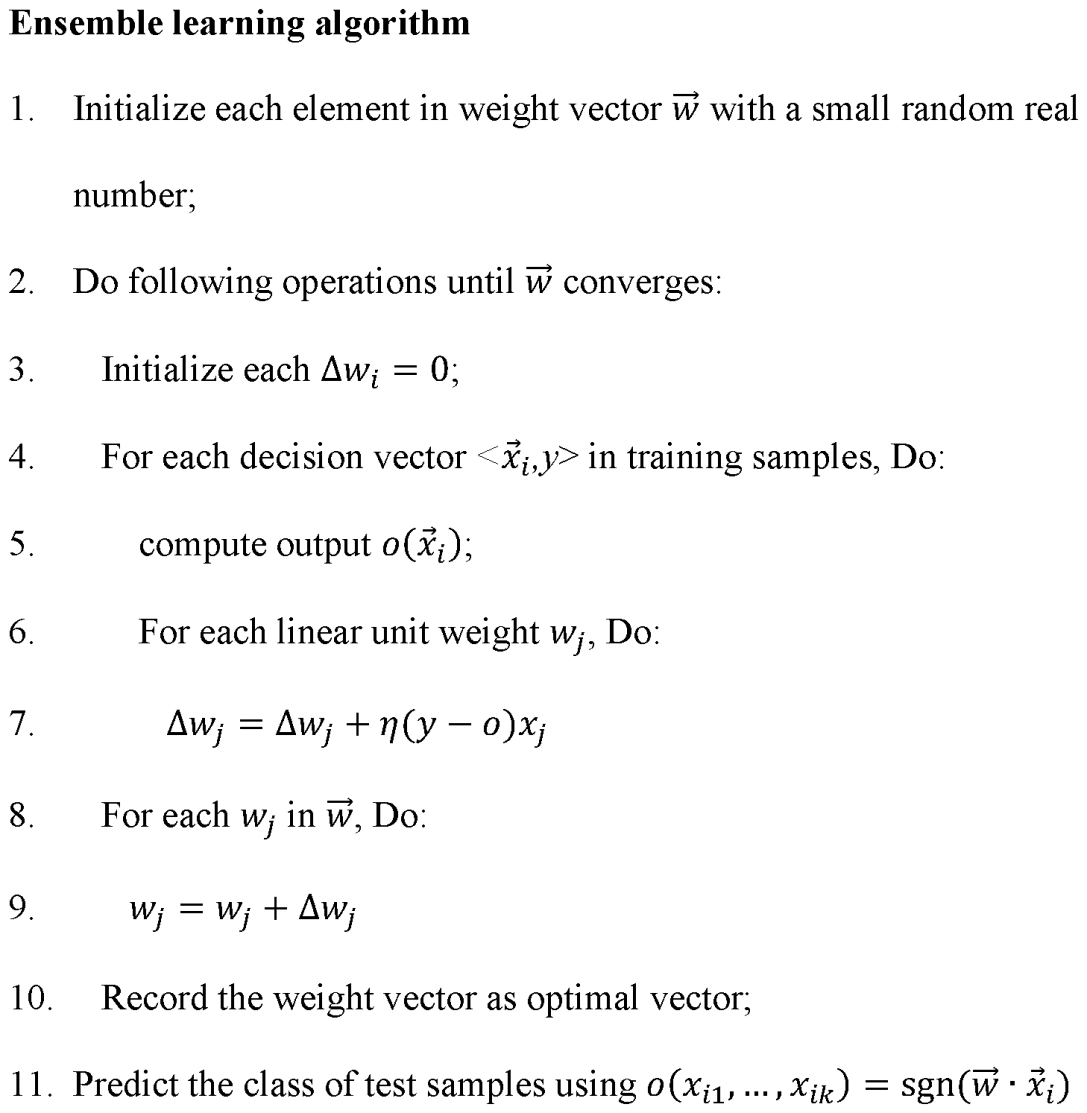
Ensemble learning algorithm.

The overall ensemble learning method is summarized in Figure 2. First, we assign an initial random weight vector for 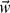. The ensemble model is then applied to all training genes and each weight is then updated by adding 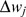 computed according to equation (17) above. This process is repeated until 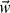 converges. Note that if *η* is a large number, the search exploration might overstep the minimum point in the error surface rather than settling into it. Therefore, the value of *η* should be gradually reduced as the number of iteration grows.

## Experimental Results

For evaluation, we benchmark our proposed EPU algorithm against four state-of-the-art techniques for disease gene prediction: PUDI method, Smalter's method, Xu's method and ProDiGe. In addition, we also compare the performance of EPU with its base learning models, namely MSVM, WKNN and WNB. Finally, we demonstrate *novel* disease gene prediction using the EPU algorithm.

### Experimental settings

We use the disease classes with at least 50 confirmed disease genes from the 21 specific disease classes in [6] for evaluating our classification algorithm. There are six such disease classes: cardiovascular disease, endocrine disease, cancer disease, metabolic disease, neurological disease, and ophthalmological disease (See Table S1 for the exact numbers of disease genes for each class). Given a particular disease class, the positive set *P* consists of all its confirmed disease genes, while the unlabeled set *U* is formed by randomly selecting from the genes that are not known to be associated with any disease such that |*P*| = |*U*|, following the setting in [9] [10] [15]. To avoid bias in sampling, we randomly select 10 groups of unlabeled set *U*. All experimental evaluations of the classification models are done on identical groups of training and test data, and we report the average performance over the 10 groups of (*P*+*U*) sets. To evaluate the performance of our algorithm, 3 fold cross validation is applied where two folds in *P+U* as the training set build classifier while remaining one fold is the test set. Next, positive training genes in *P* are used as seed nodes on multiple genetic networks to weight unlabeled training genes in *U* via flow propagation. Then, to obtain the ‘soft’ classes of training genes from component learning models, leave-one-out cross-validation (LOOCV) is used on 2 fold training samples, from which each training sample is singled out to evaluate its ‘soft’ classes by component classifiers (MSVM, WKNN and WNB), which are built on the other training samples. After LOOCV, these training samples with the ‘soft’ classes are as input data to build an ensemble learning model (as shown in Figure 2) to predict 1 fold test fold. The average results on 3×10 groups of (*P+U*) sets are reported on experimental part.

### Evaluation metrics

We use precision, recall, and F-measure to measure the performance of our classification models on each of the six disease classes. The F-measure is the harmonic mean of precision (denoted as *p*) and recall (denoted as *r*), defined as
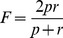
(18)


The value of F-measure is large only when both of *p* and *r* are high, and small when either of them is poor. This is appropriate for our objective of accurately predicting disease genes, as deficiencies in either precision or recall will be reflected by a low F-measure.

### Experimental Results

#### Benchmarking of EPU ensemble learning algorithm against state-of-art techniques

First, we compared our EPU algorithm against four state-of-the-art techniques, namely, PUDI method [13], Smalter's method [10], Xu's method [15] and ProDiGe [12]. Table 1 shows that our proposed EPU, on average, is 6.5%, 15.1%, 16.2% and 16.4% better than PUDI, ProDiGe, Smalter's method, Xu's method in terms of F-measure respectively. In particular, EPU can achieve much better precision and consistently better recall when compared against the recently proposed method PUDI. It shows that EPU can effectively extract hidden positive and negative data from the unlabeled data to boost classification performance.

**Table 1:** Overall comparison of classification performance among different techniques.

Disease group	Techniques	Precision (p)	Recall (r)	F-measure (F)
Cardiovascular	PUDI	82.0%	80.3%	80.4%
	ProDiGe	54.3%	96.3%	69.3%
	Smalter's method	75.4%	67.6%	70.6%
	Xu's method	72.1%	60.0%	65.4%
	EPU	85.2%	81.0%	**84.1%**
Endocrine	PUDI	83.6%	75.3%	79.2%
	ProDiGe	57.3%	87.7%	69.3%
	Smalter's method	76.4%	58.8%	66.5%
	Xu's method	75.4%	62.0%	68.0%
	EPU	88.1%	87.7%	**87.9%**
Neurological	PUDI	70.3%	80.1%	74.9%
	ProDiGe	63.1%	74.0%	68.1%
	Smalter's method	60.6%	65.9%	63.1%
	Xu's method	59.7%	66.7%	63.0%
	EPU	78.2%	80.4%	**78.6%**
Metabolic	PUDI	80.1%	84.8%	82.4%
	ProDiGe	58.7%	84.5%	69.3%
	Smalter's method	59.1%	84.7%	69.6%
	Xu's method	65.6%	78.3%	71.4%
	EPU	83.3%	93.9%	**90.9%**
Ophthalmological	PUDI	71.6%	78.5%	74.9%
	ProDiGe	58.3%	77.7%	66.6%
	Smalter's method	56.7%	77.8%	65.5%
	Xu's method	64.2%	71.3%	67.4%
	EPU	89.3%	81.0%	**84.7%**
Cancer	PUDI	76.3%	80.0%	78.0%
	ProDiGe	71.1%	79.8%	75.3%
	Smalter's method	73.8%	79.0%	76.3%
	Xu's method	71.0%	79.7%	75.1%
	EPU	81.2%	84.5%	**82.6%**
Average performance	PUDI	77.3%	79.8%	78.3%
	ProDiGe	60.5%	83.3%	69.7%
	Smalter's method	67.0%	72.3%	68.6%
	Xu's method	68.0%	69.7%	68.4%
	EPU	84.2%	84.8%	**84.8%**

PUDI is a SVM-based approach that partitions unlabeled genes into multiple levels with different associations to confirmed disease genes. ProDiGe is a bagging method that iteratively chooses random subsets from unlabeled subset and trains multiple classifiers. Smalter's method integrates multiple biological features, such as topological features, sequence-derived features, evolutionary age features. Xu's method employs the KNN classifier to predict disease genes.

#### Comparison of EPU with base classifiers

Next, we compared the performance of our proposed EPU against its base classifiers MSVM, WNB, WKNN. As shown in Table 2, on average, MSVM achieved the highest F-measure (81.3%), much higher than WNB (69.5%) and WKNN (68.7%). This is not surprising as MSVM can handle multiple weighted positive and negative sets when building its classification model. Furthermore, SVM is known to perform significantly better than NB and KNN in many real-world applications.

**Table 2:** Overall comparison to single-expert classifiers.

Disease group	Techniques	Precision (*p*)	Recall (*r*)	F-measure (*F*)
Cardiovascular	MSVM	74.3%	87.6%	80.4%
	WNB	57.3%	72.5%	63.9%
	WKNN(3)	60.1%	68.6%	64.0%
	EPU	85.2%	81.0%	**84.1%**
Endocrine	MSVM	83.4%	85.2%	84.2%
	WNB	61.3%	70.4%	65.3%
	WKNN(3)	64.5%	53.1%	57.9%
	EPU	88.1%	87.7%	**87.9%**
Neurological	MSVM	69.3%	83.7%	75.8%
	WNB	61.1%	74.4%	67.0%
	WKNN(3)	62.3%	67.1%	64.6%
	EPU	78.2%	80.4%	**78.6%**
Metabolic	MSVM	84.0%	91.3%	87.4%
	WNB	68.8%	79.9%	73.9%
	WKNN(3)	76.6%	78.8%	77.6%
	EPU	83.3%	93.9%	**90.9%**
Ophthalmological	MSVM	78.4%	86.1%	81.9%
	WNB	61.2%	78.7%	68.8%
	WKNN(3)	67.3%	72.2%	69.6%
	EPU	89.3%	81.0%	**84.7%**
Cancer	MSVM	73.4%	83.9%	78.3%
	WNB	72.5%	85.1%	78.3%
	WKNN(3)	76.4%	81.0%	78.6%
	EPU	81.2%	84.5%	**82.6%**
Average performance	MSVM	78.6%	86.3%	81.3%
	WNB	63.7%	76.8%	69.5%
	WKNN(3)	67.9%	70.1%	68.7%
	EPU	84.2%	84.8%	**84.8%**

EPU is compared with its three component classifiers Multi-level Support Vector Machine (MSVM), Weighted Naïve Bayes (WNB) and Weighted K-Nearest Neighbor (KNN) on 6 disease groups. WKNN(3) is an instance-based classifier that predicts the class of an unlabeled gene based on its 3 closest labeled genes.

Our proposed ensemble learning method EPU is able to achieve 84.8% in terms of F-measure, which is 3.5%, 15.3% and 16.1% better than MSVM, WNB and WKNN respectively. Moreover, EPU consistently outperformed all 3 component classifiers for every disease class. This strongly demonstrates that EPU can effectively integrate multiple classification models and minimize the overall error rate through dynamically assigning different weights to different classification models.

#### Sensitivity study on the parameter η in EPU and parameter k in genetic similarity networks

We perform the sensitivity study for parameter *η* in EPU and coverage of genetic similarity networks. Parameter *η* is the learning rate in EPU algorithm. We perform EPU on six disease groups with *η* from 0.001 to 0.03. The result indicates that step size within 0.001 is small enough to move optimal value point in hypothesis space and our EPU is robust and stable when *η* is small (Table S3 for the detailed results). In addition, we study the effect of the parameter *k* that determines the number of neighbors of each gene in biological networks. The results in Table S4 show that EPU consistently achieved best performance with *k* in (1, 9).

#### Comparing EPU with existing ensemble learning approaches

We also compared our proposed EPU with two existing ensemble approaches, majority vote and weighted majority vote [31], in terms of F-measure across the six disease classes. EPU was shown to outperform the existing ensemble methods (see Table S2 for the detailed results), indicating that our proposed EPU is a superior ensemble strategy for integrating multiple classification models for disease gene prediction.

#### Predicting novel disease genes for disease groups

To demonstrate novel disease gene prediction using the EPU algorithm, we selected two important disease groups, namely, metabolic and cancer, as detailed case studies. For each target disease class, we obtained a set of confirmed disease genes from OMIM and GENECARD as the positive training set, and applied our proposed EPU algorithm to prioritize a novel disease gene from the unlabeled gene set.

We first applied our EPU algorithm to discover novel disease genes for metabolic diseases. 12 unlabeled genes were detected to be associated with target disease using our algorithm. For verification, we searched the literature for evidence that supports the association of these predicted disease genes to metabolic diseases. We found that two predicted genes, RHEB and DOK5, have indeed been reported to be associated with metabolic diseases. Rheb, a GTP-binding protein, was reported to be inactivated to protect cardiomyocyte during energy deprivation via activation of autophagy. This implies that RHEB is a key regulator of autophagy during myocardial ischemia, which has implications in patients with obesity and metabolic syndrome [32]. As for DOK5, Tabassum *et al.* identified that it is a novel candidate disease genes associated with type 2 diabetes, which is a metabolic disorder due to obesity [33].

Our EPU model also predicted 32 unlabeled genes as candidate genes associated with cancer. Seven of them, SIGLEC7, PRDX4, PRDX5, HNRNPL, SRPK1, ABCB10 and PHF10 have been reported to be associated with cancer diseases. Table 3 lists these candidate disease genes and the supporting literature evidence that we have found.

**Table 3:** Novel cancer-related genes predicted by EPU.

Gene ID	Supported literatures
SUGLEC7	Ito A. et al. (2001) Binding specificity of siglec7 to disialogangliosides of renal cell carcinoma: possible role of disialogangliosides in tumor progression. FEBS Lett.
PRDX4	Lee S.U. et al. (2008) Involvement of peroxiredoxin IV in the 16alpha-hydroxyestrone-induced proliferation of human MCF-7 breast cancer cells. Cell Biol Int 32(4): 401–5.
	Park H.J. et al. (2008) Proteomic profiling of endothelial cells in human lung cancer. J Proteome Res 7(3):1138–50.
PRDX5	Enqman L., et al. (2003) Thioredoxin reductase and cancer cell growth inhibition by organotellurium compounds that could be selectively incorporated into tumor cells. Bioorg Med Chem 11(23): 5091–100.
	McNaughton M., et al. (2004) Cyclodextrin-derived diorganyl tellurides as glutathione peroxidase mimics and inhibitors of thioredoxin reductase and cancer cell growth. J Med Chem 47(1): 233–9.
	Enqman L., et al. (2000) Water-soluble organotellurium compounds inhibit thioredoxin reductase and the growth of human cancer cells. Anticancer Drug Des. 15(5): 323–30.
HNRNPL	Goehe, R.W., et al. (2010) hnRNPL regulates the tumorigenic capacity of lung cancer xenografts in mice via caspase-9 pre-mRNA processing. J. Clin. Inves. 120(11): 3923.
	Hope N.R., et al. (2011) The expression profile of RNA-binding proteins in primary and metastatic colorectal cancer: relationship of heterogeneous nuclear ribonucleoproteins with prognosis. Hum Pathol. 42(3): 393–402.
SRPK1	Hayes, G.M., et al. (2007) Serine-arginine protein kinase 1 overexpression is associated with tumorigenic imbalance in mitogen-activated protein kinase pathways in breast, colonic, and pancreatic carcinomas. Cancer Res. 67(5): 2972–80.
ABCB10 PHF10	Tang, L., et al. (2009) Exclusion of ABCB8 and ABCB10 as cancer candidate genes in acute myeloid leukemiaLetter to the Editor. Leukemia 23: 1000–2.
	Wet M., et al. (2010) Preparation of PHF10 antibody and analysis of PHF10 expression gastric cancer tissues. Journal of Xiao Bao Yu Fen Zi Mian Yi Xue 26(9): 874–6.
	Li C., et al. (2012) MicroRNA-409-3p regulates cell proliferation and apoptosis by targeting PHF10 in gastric cancer. Cancer Lett 320(2): 187–97.
SUGLEC7	Ito A. et al. (2001) Binding specificity of siglec7 to disialogangliosides of renal cell carcinoma: possible role of disialogangliosides in tumor progression. FEBS Lett.

EPU is used to discover novel cancer related genes from unlabeled gene set. The table list 12 candidate genes associated with cancer and their corresponding literature evidences.

For other candidate cancer genes without literature evidence support, seven of them, PMM1, SRCIN1, ISY1, KDM4A, CIR1, PPP2R5A and NOL3 have been shown to associate with cancer diseases in GO similarity network, GE similarity network and PPI network. From GO similarity network, PMM1 is one of top 5 nearest neighbours of cancer disease gene PPM1D and SCRIN1 is one of neighbours of disease gene CTNNB1. In GE similarity network, ISY1 is linked to disease gene P2RX7, KDM4A and CIR1 are interacted with disease genes CTNNB1 and MSH2 respectively, indicating that three suspicious genes are highly correlated with cancer disease genes in terms of gene expression. From PPI network, PPP2R5A is directly interacted with two disease genes, BCL2 and TP53, and NOL3 is linking to two disease genes, BAX and CASP8.

## Conclusions and Discussion

Despite the considerable progress in disease gene discovery, there are still many unknown disease genes that are yet to be characterized. Machine learning methods can be used to predict novel disease genes from the confirmed disease genes, based on the observation that genes associated with similar disease phenotypes are likely to share similar biological characteristics. However, there are two challenging issues for disease gene predictions. Firstly, how to leverage various biological sources during our model building process, which could effectively alleviate the bias issues from the incompleteness and noise in the data. Secondly, how to integrate multiple computational models to minimize the potential bias and errors as individual learning methods has their inherent limitations and they could predict accurately for some disease genes but could fail badly for the other ones. In this work, we have designed a novel ensemble learning method EPU for predicting disease genes via using a network-based random walk with restart approach on multiple biological networks, and an ensemble classification approach on multiple machine-learned prediction models. By using multiple biological data sources, EPU is less susceptible to potential bias, incompleteness and noise in individual data source. In this paper, we choose Nearest Neighbor, Naïve Bayes and SVM as three base learning models of EPU due to three reasons: they are the state-of-the-art learning techniques that have been widely used in disease gene identification filed [10] [11] [15] [17] [34]; 2) we are combining PU learning models instead of traditional classification models – we choose the three classification models as they can be easily adapted to build PU learning models; 3) they are quite diverse with learning criterions, so that their complementary nature may contribute a more accurate and robust combinational result. By employing an ensemble approach for prediction, EPU also minimizes the inherent limitations of individual prediction models. Finally, by employing PU learning techniques for building its ensemble of classification models, EPU is able to treat the unknown genes appropriately as an unlabeled set *U* (instead of a negative set *N*) for training, thereby resulting in more robust predictions. Experimental evaluations have confirmed the effectiveness of our proposed approach, with our EPU method consistently performing much better than the existing state-of-the-art techniques for disease gene prediction on six disease classes.

As more biological data sources and machine learning classifiers become available in the future, our EPU method can be an effective framework to integrate the additional biological and computational resources for better disease gene predictions. For further work, we will explore the inclusion of other biological data sources for disease gene prediction using our framework. Given that many machine learning problems in biomedical research do involve ensemble Positive Unlabeled data, we can also adapt our EPU framework to other applications, such as drug-target interaction prediction [35] [36].

## Supporting Information

Figure S1 Procedure of extracting candidate positive set.
(DOCX)

Table S1 Number of disease genes associated with six disease classes.
(DOCX)

Table S2 Performance comparison of ensemble methods.
(DOCX)

Table S3 Effect of parameter η on classification performance of six disease groups.
(DOCX)

Table S4 Sensitive analysis on biological network noise to disease gene prediction.
(DOCX)
