# Supplementary material for: Ensemble Positive Unlabeled Learning for Disease Gene Identification
Source: PLoS One. 2014 May 9;9(5):e97079. doi: 10.1371/journal.pone.0097079 (PMC4016241; doi:10.1371/journal.pone.0097079)
Supplement: Figure S1 — Procedure of extracting candidate positive set. (DOCX) [file pone.0097079.s001.docx]

**
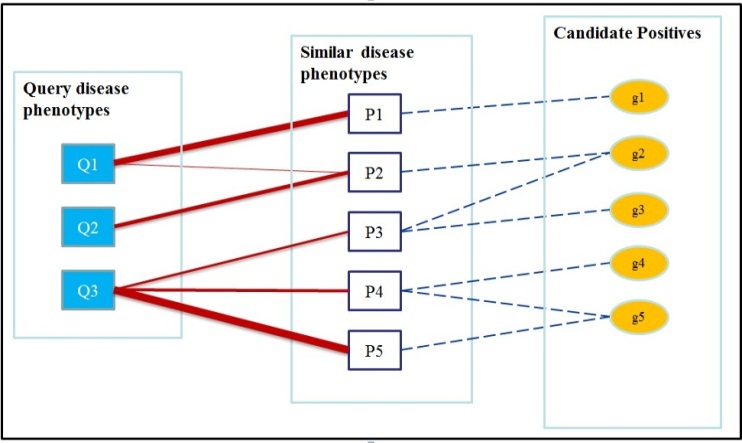
**

**Figure S1.** **Procedure of extracting candidate positive set**. Figure S1 illustrates this procedure with an example. Q1-Q3 are phenotypes associated with a query disease class (e.g. Cancer). Similar phenotypes, denoted by P1-P5, are identified using the Phenotype similarity network based on their similarity with the query disease phenotypes. For example, Q1 and P1, Q2 and P2, and Q3 and P5 are highly similar phenotypes (thicker lines represent higher similarity) based on the phenotype similarity network that we have constructed earlier. The known disease genes (denoted here by g1-g5) associated with P1-P5 are then used to form the set of candidate positive genes.
