# Supplementary material for: Ensemble Positive Unlabeled Learning for Disease Gene Identification
Source: PLoS One. 2014 May 9;9(5):e97079. doi: 10.1371/journal.pone.0097079 (PMC4016241; doi:10.1371/journal.pone.0097079)
Supplement: Table S1 — Number of disease genes associated with six disease classes. (DOCX) [file pone.0097079.s002.docx]

**Table S1. Number of disease genes associated with six disease classes**.

| **Disease Class** | **Cardiovascular** | **Endocrine** | **Metabolic** | **Neurological** | **Ophthalmological** | **Cancer** |
| --- | --- | --- | --- | --- | --- | --- |
| No. of genes | 107 | 81 | 263 | 217 | 163 | 210 |
