# Supplementary material for: Ensemble Positive Unlabeled Learning for Disease Gene Identification
Source: PLoS One. 2014 May 9;9(5):e97079. doi: 10.1371/journal.pone.0097079 (PMC4016241; doi:10.1371/journal.pone.0097079)
Supplement: Table S2 — Performance comparison of ensemble methods. (DOCX) [file pone.0097079.s003.docx]

**Table S2.** **Performance comparison of ensemble methods**. We compare our EPU with two ensemble baselines: the first one is to adopt a majority vote of the three models trained individually; the second one applies a weighted majority vote based on accuracy of component models. Table S2 performs evaluation of three ensemble approaches on six disease groups, and EPU consistently outperforms other ensemble methods significantly, which indicates neither two existing combination baselines is able to balance component classifiers with proper weights. Majority vote has the worst performance due to equally weighting all components for any disease group evaluations. On the other hand, weighted majority vote roughly equates single classifier scenario with that in ensemble classifiers. Unlike above two approaches, EPU uses Gradient decent to optimize the weights of each component classifiers under each disease group, which regulates the weights with respect to different disease groups.

| **Disease group** | **Techniques** | **Precision (*p*)** | **Recall (*r*)** | **F-measure (*F*)** |
| --- | --- | --- | --- | --- |
| Cardiovascular | Weighted majority vote | 73.7% | 87.3% | 80.0% |
|  | majority vote | 56.3% | 80.0% | 66.0% |
|  | EPU | 85.2% | 81.0% | **84.1%** |
| Endocrine | Weighted majority vote | 86.1% | 84.0% | 85.0% |
|  | majority vote | 65.5% | 73.3% | 67.9% |
|  | EPU | 88.1% | 87.7% | **87.9%** |
| Neurological | Weighted majority vote | 69.6% | 83.5% | 75.9% |
|  | majority vote | 65.3% | 74.7% | 70.0% |
|  | EPU | 78.2% | 80.4% | **78.6%** |
| Metabolic | Weighted majority vote | 86.6% | 92.5% | 89.5% |
|  | majority vote | 68.4% | 89.1% | 77.4% |
|  | EPU | 83.3% | 93.9% | **90.9%** |
| Ophthalmological | Weighted majority vote | 76.9% | 87.3% | 81.8% |
|  | majority vote | 59.4% | 78.7% | 67.7% |
|  | EPU | 89.3% | 81.0% | **84.7%** |
| Cancer | Weighted majority vote | 78.7% | 80.3% | 79.5% |
|  | majority vote | 69.7% | 93.7% | 79.9% |
|  | EPU | 81.2% | 84.5% | **82.6%** |
| Average performance | Weighted majority vote | 78.5% | 86.0% | 81.8% |
|  | majority vote | 64.1% | 81.6% | 71.5% |
|  | EPU | 84.2% | 84.8% | **84.8%** |
