# Supplementary material for: Ensemble Positive Unlabeled Learning for Disease Gene Identification
Source: PLoS One. 2014 May 9;9(5):e97079. doi: 10.1371/journal.pone.0097079 (PMC4016241; doi:10.1371/journal.pone.0097079)
Supplement: Table S3 — Effect of parameter η on classification performance of six disease groups. (DOCX) [file pone.0097079.s004.docx]

**Table S3.** **Effect of parameter *η* to classification performance on six disease groups**. We perform a sensitivity study for the parameter *η* used in the algorithm. Parameter *η* is the learning rate for optimizing the linear combination of prediction. To study its effect, we run our EPU with *η* from 0.001 to 0.03 in the scales of 0.005. The performance is measured on six disease groups using three fold cross validation. Results are shown in Table S3. The F-measure is relevant steady with the value of *η* from 0.0005 to 0.001, indicating that step size is small enough to move the area of minimum points in hypothesis space. However, if we further increase the step size of learning rate, the exploration might cross the optimal (minimum) value point in hypothesis space, instead of staying that point, the searched large weight vector eventually affects the performance of our ensemble algorithm. Nevertheless, the un-stable result with *η* from 0.005 to 0.03 suggests that our algorithm is robust and steady when *η* becomes small.

| **Disease Group** | **Parameter** *η* | **Precision (*p*)** | **Recall (*r*)** | **F-measure (*F*)** |
| --- | --- | --- | --- | --- |
| Cardiovascular | 0.0005 | 85.2% | 81.0% | 84.0% |
|  | 0.001 | 84.4% | 81.9% | **84.1%** |
|  | 0.005 | 89.5% | 76.2% | 81.9% |
|  | 0.01 | 88.8% | 76.2% | 81.3% |
|  | 0.015 | 89.4% | 74.3% | 80.4% |
|  | 0.025 | 86.7% | 81.0% | 83.2% |
|  | 0.03 | 90.9% | 74.3% | 80.9% |
| Endocrine | 0.0005 | 88.8% | 81.1% | 84.5% |
|  | 0.001 | 87.3% | 83.3% | **84.9%** |
|  | 0.005 | 92.2% | 77.8% | 83.7% |
|  | 0.01 | 91.0% | 80.0% | 84.7% |
|  | 0.015 | 90.4% | 78.9% | 84.1% |
|  | 0.025 | 90.4% | 78.9% | 84.1% |
|  | 0.03 | 90.4% | 78.9% | 84.1% |
| Neurological | 0.0005 | 82.0% | 76.7% | 78.3% |
|  | 0.001 | 77.7% | 81.1% | **79.2%** |
|  | 0.005 | 81.9% | 76.3% | 78.1% |
|  | 0.01 | 82.1% | 75.9% | 78.0% |
|  | 0.015 | 82.0% | 75.5% | 77.7% |
|  | 0.025 | 81.3% | 77.5% | 78.5% |
|  | 0.03 | 79.4% | 77.5% | 78.0% |
| Metabolic | 0.0005 | 89.0% | 92.5% | 90.6% |
|  | 0.001 | 89.0% | 92.9% | **90.8%** |
|  | 0.005 | 88.7% | 92.9% | 90.6% |
|  | 0.01 | 88.7% | 92.9% | 90.6% |
|  | 0.015 | 89.3% | 92.1% | 90.6% |
|  | 0.025 | 88.4% | 93.6% | 90.9% |
|  | 0.03 | 88.7% | 93.3% | 90.8% |
| Ophthalmological | 0.0005 | 90.4% | 86.1% | 88.1% |
|  | 0.001 | 90.4% | 86.1% | **88.1%** |
|  | 0.005 | 89.5% | 85.2% | 87.2% |
|  | 0.01 | 90.4% | 85.2% | 87.6% |
|  | 0.015 | 90.4% | 85.2% | 87.6% |
|  | 0.025 | 90.3% | 84.3% | 87.0% |
|  | 0.03 | 90.3% | 84.3% | 87.0% |
| Cancer | 0.0005 | 83.9% | 78.5% | 81.0% |
|  | 0.001 | 82.4% | 81.9% | 82.1% |
|  | 0.005 | 83.3% | 80.8% | 81.9% |
|  | 0.01 | 83.1% | 80.2% | 81.6% |
|  | 0.015 | 82.4% | 81.9% | 82.1% |
|  | 0.025 | 82.3% | 81.4% | 81.7% |
|  | 0.03 | 82.5% | 82.5% | **82.4%** |
