# Supplementary material for: Ensemble Positive Unlabeled Learning for Disease Gene Identification
Source: PLoS One. 2014 May 9;9(5):e97079. doi: 10.1371/journal.pone.0097079 (PMC4016241; doi:10.1371/journal.pone.0097079)
Supplement: Table S4 — Sensitive analysis on biological network noise to disease gene prediction. (DOCX) [file pone.0097079.s005.docx]

**Table S4.** **Sensitive analysis on biological network noise to disease gene prediction**. We conduct experiments for sensitivity analysis on coverage of biological networks. Table S4 studies the effect of the parameter *k* that decides the number of direct neighbors linking each gene in PPI network, GO similarity network and gene expression similarity network. We ran EPU with *k* from 1 to 9 with fix value *η* = 0.001. Table S4 indicates the performance on 6 disease groups. For disease groups Neurological, Metabolic and Ophthalmological, the performance of EPU algorithm did improve with increasing value of *k* from 1 to 5, indicating that incorporating more informative similar neighbors is helpful for prioritizing disease genes. However, if we further include more neighbors (e.g. when *k* > 8) with low genetic similarities, noisy and un-meaningful neighbors will be included and eventually affects the performance of disease gene prediction. For example, the results in Cardiovascular and Neurological disease groups showed that the performance with *k* = 9 has worsened. Finally we observed in table that not all disease groups achieved best performance in range of (1, 9). For example, the performance on cancer disease remained improved with increasing value of *k*, indicating that cancer disease has different pathogenic rules from others. Nevertheless, the performance of EPU algorithm with *k* in wide range was consistently better than that of PUDI and ProDiGe, suggesting that EPU is insensitive to the specific value of *k*.

| **Disease group** | **KNN (*k*)** | **F-measure (*F*)** |
| --- | --- | --- |
| Cardiovascular | 1 | 84.1% |
|  | 2 | 83.8% |
|  | 3 | 82.3% |
|  | 4 | 82.9% |
|  | 5 | 82.3% |
|  | 6 | 82.0% |
|  | 7 | 82.6% |
|  | 8 | 82.6% |
|  | 9 | 82.6% |
| Endocrine | 1 | 87.1% |
|  | 2 | 85.2% |
|  | 3 | 87.3% |
|  | 4 | 87.1% |
|  | 5 | 87.1% |
|  | 6 | 87.9% |
|  | 7 | 87.9% |
|  | 8 | 87.9% |
|  | 9 | 87.9% |
| Neurological | 1 | 75.0% |
|  | 2 | 75.2% |
|  | 3 | 75.1% |
|  | 4 | 75.2% |
|  | 5 | 78.0% |
|  | 6 | 75.7% |
|  | 7 | 75.9% |
|  | 8 | 76.6% |
|  | 9 | 76.0% |
| Metabolic | 1 | 90/1% |
|  | 2 | 89.4% |
|  | 3 | 89.8% |
|  | 4 | 90.5% |
|  | 5 | 90.9% |
|  | 6 | 90.5% |
|  | 7 | 90.5% |
|  | 8 | 90.1% |
|  | 9 | 89.9% |
| Ophthalmological | 1 | 83.0% |
|  | 2 | 83.0% |
|  | 3 | 84.0% |
|  | 4 | 84.0% |
|  | 5 | 83.6% |
|  | 6 | 83.6% |
|  | 7 | 84.0% |
|  | 8 | 84.0% |
|  | 9 | 83.2% |
| Cancer | 1 | 81.8% |
|  | 2 | 81.4% |
|  | 3 | 80.8% |
|  | 4 | 82.2% |
|  | 5 | 81.2% |
|  | 6 | 81.7% |
|  | 7 | 82.2% |
|  | 8 | 82.2% |
|  | 9 | 82.4% |
